# Poor Nutrition Status and Associated Feeding Practices among HIV-Positive Children in a Food Secure Region in Tanzania: A Call for Tailored Nutrition Training

**DOI:** 10.1371/journal.pone.0098308

**Published:** 2014-05-20

**Authors:** Bruno F. Sunguya, Krishna C. Poudel, Linda B. Mlunde, David P. Urassa, Junko Yasuoka, Masamine Jimba

**Affiliations:** 1 Department of Community and Global Health, Graduate School of Medicine, The University of Tokyo, Tokyo, Japan; 2 Department of Public Health, School of Public Health and Health Sciences, University of Massachusetts Amherst, Amherst, Massachusetts, United States of America; 3 School of Public Health and Social Sciences, Muhimbili University of Health and Allied Sciences, Dar es Salaam, Tanzania; University of Washington, United States of America

## Abstract

Undernutrition among HIV-positive children can be ameliorated if they are given adequate foods in the right frequency and diversity. Food insecurity is known to undermine such efforts, but even in food rich areas, people have undernutrition. As yet no study has examined feeding practices and their associations with nutrition status among as HIV-positive children in regions with high food production. We therefore examined the magnitude of undernutrition and its association with feeding practices among HIV-positive children in a high food production region in Tanzania.

**Methods:**

We conducted this mixed-method study among 748 children aged 6 months-14 years attending 9 of a total of 32 care and treatment centers in Tanga region, Tanzania. We collected quantitative data using a standard questionnaire and qualitative data through seven focus group discussions (FGDs).

**Results:**

HIV-positive children had high magnitudes of undernutrition. Stunting, underweight, wasting, and thinness were prevalent among 61.9%, 38.7%, 26.0%, and 21.1% of HIV-positive children, respectively. They also had poor feeding practices: 88.1% were fed at a frequency below the recommendations, and 62.3% had a low level of dietary diversity. Lower feeding frequency was associated with stunting (β = 0.11, p = 0.016); underweight (β = 0.12, p = 0.029); and thinness (β = 0.11, p = 0.026). Lower feeding frequency was associated with low wealth index (β = 0.06, p<0.001), food insecurity (β = −0.05, p<0.001), and caregiver's education. In the FGDs, participants discussed the causal relationships among the key associations; undernutrition was mainly due to low feeding frequency and dietary diversity. Such poor feeding practices resulted from poor nutrition knowledge, food insecurity, low income, and poverty.

**Conclusion:**

Feeding practices and nutrition status were poor among HIV-positive children even in food rich areas. Improving feeding frequency may help to ameliorate undernutrition. To improve it, tailored interventions should target children of poor households, the food insecure, and caregivers who have received only a low level of education.

## Background

Poor feeding practices undermine efforts to combat unacceptably high rates of undernutrition among children in developing countries [1], [2]. Such practices include low feeding frequency, low dietary diversity, inadequate quantity, and diets with poor quality [1], [3]. Efforts to improve feeding practices can also improve undernutrition among children in developing countries [4]–[7].

Poverty and other socio-demographic disadvantages can limit adequate feeding practices [8], [9]. Households in low economic strata are prone to food insecurity [10]–[12]. Under such circumstance, poor households have limited choices for food with adequate nutritional values [10], [12]. They usually adapt themselves to this situation by cutting down the number of basic meals or reducing the amount in each meal [13], [14]. In a typical household affected by food insecurity, children are usually less afflicted during its early stage. At this stage, other members of the household tend to reduce amount and frequency of food for themselves for the sake of children. However, children are more likely to be affected by extreme forms of food insecurity and later, hunger. Socio-economic disadvantages and the cycle of poverty are also associated with poor education. Children of poorly educated caregivers succumb to various forms of undernutrition [15]. The possible link could be through poor feeding practices [16].

Through high agricultural yield or high purchasing power, food availability can reduce a household's food insecurity [17]. However, even when sufficient food is available, its consumption may not be adequate. This is because the consumption of diverse types of foods in adequate quality, quantity, and frequency may depend on nutrition knowledge of caregivers [3], [18]. This may also be a reason behind the high rates of undernutrition among children in the general populations of many developing countries, including Tanzania, even where food productivity is high [19], [20].

In developing countries, if children are affected by HIV/AIDS in a household, such household is more likely to have lower potential to provide them with food of adequate quality, quantity, and in the required frequency [14]–[16], [21]. Furthermore, HIV-positive children have special nutritional needs, different from their HIV-negative counterparts [22]. The World Health Organization (WHO) recommends a 10% increase in energy intake for the HIV-positive child growing well on antiretroviral therapy (ART) above the normal requirement of an otherwise HIV-negative child of the same age [22]. A 20–30% increase in energy intake is required to sustain an HIV-positive child with HIV-related symptoms including TB, chronic lung infections, or persistent diarrhea [22]. HIV-positive children need an extra 50–100% of energy intake compared to an otherwise normal child if they have severe malnutrition or severe failure to thrive, regardless of their ART status [22]. These energy requirements are supposed to be met from the foods consumed daily. To achieve adequate nutrition, an HIV-positive child is supposed to eat at least five times a day [22]. Such meals have to be balanced, diverse, and adequate in amount.

Food insecurity and poor feeding practices can also affect the effectiveness of ART and drive the HIV-positive children further into undernutrition. Appropriate use of ART should reduce the risk of a child succumbing to opportunistic infections that can affect his/her nutrition status [23]–[25]. However, its use comes with its own risks especially in the context of food insecurity and hunger [26]. Food insecurity is known to intensify ART's side effects to the extent of intolerability [21]. To avoid symptoms of ART's side effects, caregivers in some areas give their children ART when they are sure of accessing food to accompany it [27]. This results in poor adherence and sometimes pushing the children further into advanced stages of HIV and undernutrition [21], [26]. In Tanzania, too, households with HIV-positive children had a lower dietary diversity score, feeding frequency, and a higher proportion of food insecurity compared to households with HIV-negative children [28].

To decrease undernutrition among HIV-positive children in Tanzania and in other similar areas, feeding practices should be improved [28]. However, to provide culturally appropriate interventions, it is important to understand the local determinants of poor feeding practices among such children. So far, little has been examined about the determinants of poor feeding practices among HIV-positive children in areas where food production is high. In this study, we therefore first examined the magnitude of undernutrition and poor feeding practices. Next, we examined local determinants of undernutrition including its association with feeding practices. Third, we examined the local factors associated with poor feeding practices, in particular, low feeding frequency, among children living with HIV/AIDS in Tanga, Tanzania. Finally, we further explored the key associations by conducting a qualitative inquiry.

## Methods

### Study design and area

We employed a mixed method design for this study. First, we conducted a cross-sectional quantitative study to examine the magnitude and determinants of undernutrition and poor feeding practices among HIV-positive children in Tanga, Tanzania. Results of this study guided a qualitative study. Through seven focus group discussions (FGDs), we explored possible explanations of the findings and the key associations between various determinants and undernutrition among HIV-positive children. Finally, we triangulated results from both methods to help explain the causal relations of the associations between feeding practices and nutrition statuses among HIV-positive children in this food rich region. This study seeks to contribute to the operational research [29], aiming to improve feeding practices and thus nutritional status of such vulnerable children.

In the Tanga region, a vast diversity of food is available and grown. It is the leading region in fruit and vegetable production in the country and supplies other regions with cereal, fruits, marine, and diary products. The presence of such quantity and diversity of foods is not correlated with consumption and nutrition statuses. Only 59.4% of 292 sampled under-five children in Tanga consumed foods rich in Vitamin A [19]. Tanga has the worst nutrition outcomes in the country despite the foods available. For example, stunting prevalence was the highest in the country with about 49% of 315 under-five sampled children, 12% were underweight, and 5.5% had wasting. Poor feeding practices could also be behind poor micronutrient markers among children. In the same population, 38.9% of children had Vitamin A deficiency, 36.5% had iron deficiency, and 52.2% had iron deficiency anemia [19].

A total of 20,773 people living with HIV/AIDS were enrolled in care and treatment centers (CTCs) in the Tanga region in 2009. Although the magnitude of HIV/AIDS among children has not been reported for Tanga, about 1,800 HIV-positive children have been enrolled in the CTCs for care and treatment. The current study was a hospital-based study and conducted among participants who were attending CTCs in the region. We described the CTCs' organization and distribution in a separate article [29].

After obtaining confirmatory test results using the standard algorithm [30], HIV-positive children are usually enrolled in the CTCs to receive care. Such care includes treatment with Highly Active Antiretroviral Therapy (HAART), follow up, adherence counseling, and treatment of other associated opportunistic infections [30]. They are also supposed to receive nutrition care and monitoring. However, during this study period, no specific nutrition intervention was carried out targeting HIV-positive children attending these CTCs. Health workers attending these children also were not equipped with any inservice nutrition training to improve their management skills, and the new WHO *Guidelines for an integrated approach to the nutritional care of HIV-infected children (6 months – 14 years)* [22] had not yet been locally adapted.

### Participants

We recruited pairs of HIV-positive children and their caregivers who were attending the CTCs for the child to receive care and/or treatment. Inclusion criteria were children aged between six months and fourteen years, who are registered at or have been transferred to the CTC, who have ART records, and whose caregiver gave consent to participate in the study. We excluded children with missing medical records.

We selected a convenience sample of nine out of 32 CTCs that give care to such patients. The selected health facilities represented most districts of the Tanga region and have at least 20 children registered. Out of 32 CTCs, 15 fulfilled this criterion. We selected the CTC with the highest number of children if the district had two or more CTCs with at least 20 children. Because of its size and high number of patients, we selected three CTCs from the Tanga municipality. This process resulted in a final selection of nine CTCs for this study which, according to medical records, had a total of 1,248 children registered. We recruited a total of 797 pairs of children and their caregivers for this study, who attended the clinic on the day of data collection, and fulfilled the criteria. We excluded the data of 49 children after data collection. Among them, 41 had missing variables or erroneous entries for the outcome variable, and eight had not met the selection criteria.

We also conducted seven FGD sessions with the caregivers of HIV-positive children attending CTCs in Tanga region. The initial plan was to conduct nine focus group discussions; one from each CTC selected for the quantitative survey. However, we had reached the saturation point at the seventh group and no new information was emerging. We conducted each FGD in a separate district to reach participants in different geographic areas within the region. We conveniently selected participants who agreed to participate and who had time for the discussions when they were done with care and treatment or an ART refill for their HIV-positive children. We invited eight participants for each group, for a total of 56 caregivers.

### Measurements

The outcome variables of interest were measures of nutrition status including stunting, underweight, thinness, and wasting. A low height for age, below or equal to −2 SD of the reference population, signifies moderate stunting while below or equal to −3 SD signifies severe stunting [31]–[33]. To measure height, we used a standard measuring board calibrated in centimeters and converted it to height-for-age z-scores (HAZ).

Wasting is measured by weight for height of children aged 6–60 months. A low weight for height, below or equal to −2 SD of the reference population, signifies acute moderate wasting, while below or equal to −3 SD signifies severe wasting [31]–[33]. We measured weight in grams using a standard Salter scale with minimal clothing and converted them into weight-for-height z-scores.

We also used underweight as a measure of weight for age of children up to 10 years of age [34]. A child with a weight-for-age z score below or equal to −2 SD of the reference population is moderately underweight while a child below or equal to −3 SD is severely underweight. Finally, we used BMI-z scores as a measure of thinness among children aged above five years. A BMI-z score below or equal to −2SD was considered moderate thinness, while below or equal to −3SD is severe thinness. All anthropometric calculations used WHO Anthro version 3.22 [34].

To measure feeding practices, we assessed the most common measures, which are feeding frequency and dietary diversity. In this study, we assessed feeding frequency and dietary diversity separately.

We assessed feeding frequency by asking the caregivers to recall the number of times they fed their children in the previous 24 hours. This measurement was also used in previous studies in Tanzania among HIV-positive children [35], [36]. We considered a feeding frequency below five to be a low feeding frequency. The WHO recommends a feeding frequency of at least five times a day for HIV-positive children [22], [37].

We measured dietary diversity using the dietary diversity scale (DDS) [38]. The scale is made of common food items usually found in the area of interest. We extracted a list of twelve food items from the Tanzania Demographic and Health Survey [20], [39]. In our questionnaire, we asked caregivers about the food items they had given to their children in the previous 24 hours and summed them to make the total dietary diversity score. To establish the cutoff point for low dietary diversity, we used the median value, which was three. This value also coincided with the mean DDS of the lowest two terciles of the DDS score as recommended by the developer [38]. Therefore, a DDS score of three or below was considered low DDS. The DDS scale was also used in previous studies of HIV-positive children in Tanzania [28], [36].

We assessed food insecurity using the household food insecurity access scale (HFIAS) [40]. HFIAS is a nine-item questionnaire used to measure the severity of food insecurity and household access to food according to a Likert scale. Options in this scale are 0  =  ‘no’, 1  = ’rarely’, 2  =  ‘sometimes’, and 3  =  ‘often’. The lowest total score is 0 and the highest is 27. The scale can be used as a continuous variable or categorized into four groups: food secure, mildly insecure, moderately insecure, and severely insecure. HFIAS uses a recall period of 30 days and has been used in several studies within the region [2], [41]–[45]. In this study, HFIAS had a Cronbach's alpha of 0.96, an item-rest correlation ranging from 0.63 to 0.89, and an average variance of 0.36.

To determine HIV/AIDS progression, we used the WHO clinical staging system, a four-stage classification system. We classified the first two stages as “early stages” and clinical HIV stages 3 and 4 as “advanced stages” [46]. We retrieved the data on HIV stage from the medical files of each patient. The health workers update the clinical stage on a routine basis with each patient visit to the CTC.

We measured adherence to ART using a four-day recall. We asked patients whether they took their medications, when during the day, how many times each day, and how many pills each time. Based on the HAART regimen, doctors can prescribe a once or twice a day dosage. For the twice a day dosage regimen, we considered missing one dose in four days as low adherence. For the once a day dosage regimen, we considered missing one in four day regimen as low adherence.

To assess the burden of common opportunistic infections, we asked the caregivers whether their children had been affected by tuberculosis (TB), malaria, acute respiratory infections, or diarrhea in the past six months. As in previous studies [28], [47], we defined diarrhea as the presence of three or more watery stools during the previous 24 hours. We defined malaria as a febrile illness with symptoms such as fever, chills and sweating, confirmed with laboratory investigation [48].

As in previous studies [28], [36] and other population surveys in Tanzania [20], [39], [49], we used a weighted wealth index to assess the economic status of households with HIV-positive children. The wealth index was calculated based on ownership of household durable assets. Factor analysis was used to reduce the constructed dichotomous variables from 59 to 22, which loaded as factor 1. We treated the factor loadings as item weights summed to give the wealth index for each household like in the previous studies [50]–[52].

We adopted other socio-demographic variables from the women and household questionnaires of the TDHS [20], [39]. The population surveys tested and used such variables in both 2005 and 2010 in Tanzania. Such variables included caregivers' education level, child's orphanhood state, sex, and age. We defined a caregiver as being a child's parent, relative, guardian, or anyone else above 18 years old who takes care of the child and supervises their treatment or accompanies them to the CTCs [28]. We measured education level according to the education levels in Tanzania and divided them into three levels: no formal education, having a primary level education, and above primary level, including secondary, college, and others. A child who lost one or both parents was regarded as an orphan [20], [39].

### Data collection

We collected data using a pre-tested questionnaire. We conducted a one-day training of two research assistants from each CTC on interview techniques, questionnaire content, and ethics in data collection. We collected data through face-to-face interviews and retrieved medical data from medical records from April to May 2013.

For the focus group discussions, we used an IC recorder to record the participants' discussions. Before each discussion, participants introduced their age, occupation, marital status, and number of children they care for. The moderator, who is the first author, introduced the study, explained its aims, and set ground rules. The stem questions asked by the moderator sought to find the local factors associated with undernutrition in the region despite its known vast food production, participants' feeding practices, food access, the role of nutrition education, occurrence of opportunistic infections, and local challenges encountered when feeding HIV-positive children in each district. Finally, participants were given opportunities to suggest their local solutions and what they needed from the health workers to combat poor feeding practices and undernutrition among HIV-positive children and those of the general population. All discussions were conducted in Swahili language. The conduct and reporting of the FGDs was in accordance with the consolidated criteria for reporting qualitative research (COREQ) [53].

### Data analysis

We conducted analysis using both descriptive and regression methods. For descriptive analyses, we used Chi-square tests to compare the characteristics of participants. A similar method was used to examine the magnitude of undernutrition and feeding characteristics presented in categorical variables while comparing them between males and females. We conducted bivariate linear regression to examine association between independent variables and dependent variables. In this case, we examined factors associated with each type of undernutrition. Statistical associations with p-values below or equal to 0.2 were entered into a multivariate regression to find factors associated with undernutrition after adjusting for confounders and important covariates [54].

Feeding frequency was low and associated with most of the nutrition status measures. Therefore a separate model was built to examine factors associated with feeding frequency among HIV-positive children attending CTCs in Tanga region. For this model, independent variables included sex; household wealth index; caregivers' education level; orphanhood; household food insecurity access score; acute respiratory infection or diarrhea in the past six months; and HAART adherence.

In both models, we examined multicollinearity using variance-inflating factors. None of the independent variables had a VIF value above 10 or below 0.1. We set the statistical significance at *p-value*<0.05. We conducted all analyses using STATA version 12.

A research assistant transcribed the recorded focus group discussions into Swahili scripts. A separate research assistant then translated each of the Swahili scripts into English. A local expert on qualitative research checked for quality of each step. We analyzed the transcribed text based on the themes that emerged from the discussion [55].

When the main study and all publications are completed, the data collected in this study will be made available to other researchers upon request. Data will be coded to ensure confidentiality and anonymity.

### Ethics statement

We obtained written consent from participants before the interviews and focus group discussion. Participants were assured of confidentiality and anonymity throughout the process and for all reports and publications generated. Participation was voluntary and there were no implications for care at the CTC upon refusal to participate. This study was approved by the Research Ethics Committee of the University of Tokyo, and the Expedited Review Sub-committee of the Senate Research and Publication Directorate of the Muhimbili University of Health and Allied Sciences.

## Results

### Quantitative study

#### General characteristics

We recruited 63.8% of all HIV-positive children attending the nine selected CTCs in Tanga region. Data were available for analyses from 748 pairs of HIV-positive children and their caregivers who were attending CTCs. Of the 748 HIV-positive children in this study, 666 (89.0%) were on HAART. Of those on treatment, 85.3% had high adherence to the treatment. Of all children included in this study, 69.6% were in the advanced HIV clinical stage. Among the 666 HIV-positive children on HAART, 75.7% were in the advanced HIV clinical stage. In this respect, no differences were observed between male and female participants. Table 1 shows that two thirds (66.7%) of the children had lost at least one parent. A high proportion of their caregivers (22.1%) had no formal education while only 7.6% of all caregivers had at least secondary education.

**Table 1:** Descriptive characteristics of HIV-positive children attending CTCs in Tanga.

Variable	Total		Male		Female		p-value
	n	%	n	%	n	%	
**Age (months)**							
6–59	152	20.3	81	23.7	71	17.5	0.105
60–143	422	56.4	183	53.5	239	58.9	
144–168	174	23.3	78	22.8	96	23.6	
**Parental status**							
Non-orphan	249	33.3	122	35.7	127	31.3	0.204
Orphan	499	66.7	220	64.3	279	68.7	
**Caregiver education**							
None	165	22.1	73	21.3	92	22.7	0.852
Primary	526	70.3	244	71.4	282	69.4	
>Primary	57	7.6	25	7.3	32	7.9	
**Wealth index**							
High	251	33.6	123	36.0	128	31.5	0.327
Middle	248	33.2	105	30.7	143	35.2	
Low	249	33.2	114	33.3	135	33.3	
**ART status**							
Yes	666	89.0	303	88.6	363	89.4	0.490
No	82	11.0	39	11.4	43	10.6	
**HIV stage**							
Early	227	30.4	101	29.5	126	31.0	0.656
Advanced	521	69.6	241	70.5	280	69.0	
**ART adherence**							
Low	98	14.7	49	16.1	49	13.5	0.349
High	568	85.3	255	83.9	313	86.5	
**Household food security**							
Food secure	146	19.6	70	20.5	76	18.8	0.899
Mild insecure	46	6.2	22	6.5	24	5.9	
Moderate insecure	112	15.0	49	14.4	63	15.6	
Severe insecure	442	59.2	200	58.6	242	59.7	

CTC- Care and treatment center; ART- Antiretroviral therapy; HIV- Human immunodeficiency virus.

#### Feeding practices and nutrition status of HIV-positive children in Tanga

HIV-positive children in this study had a mean dietary diversity score of 3.3 (SD 1.3) and a mean feeding frequency of 3.2 (SD 1.0). The WHO's standard recommended feeding frequency for this population is ideally five times a day. As shown in Table 2, a high proportion of HIV-positive children (88.1%) were fed at a low frequency. Feeding frequency did not differ between male and female participants. A high proportion of HIV-positive children (62.3%) had a low dietary diversity score.

**Table 2:** Magnitude of poor feeding practices and nutrition status.

Nutrition status	Male		Female		Total		p-value
	n	%	n	%	N	%	
**Feeding Frequency**							
Low	298	87.1	361	88.9	659	88.1	0.454
High	44	12.9	45	11.1	89	11.9	
**Dietary Diversity Score**							
Low	220	64.3	246	60.6	466	62.3	0.293
High	122	39.7	160	39.4	282	37.7	
**Wasting (**≤ **60 months)**							
Wasted (WHZ ≤−2SD)	29	30.2	18	21.2	47	26.0	0.167
Normal (WHZ>−2SD)	67	69.8	67	78.8	134	74.0	
**Thinness (>60 months)**							
Thin (BMIZ ≤−2SD)	56	23.1	62	19.6	118	21.1	0.316
Normal (BMIZ>−2SD)	187	76.9	255	80.4	442	78.9	
**Underweight (**≤**120 months)**							
Underweight (WAZ≤−2SD)	95	40.6	103	37.1	198	38.7	0.412
Normal (WAZ>−2SD)	139	59.4	175	62.9	314	61.3	
**Stunting (6 months-14 years)**							
Stunted (HAZ≤−2SD)	208	60.8	255	62.8	463	61.9	0.577
Normal (HAZ>−2SD)	134	39.2	151	37.2	285	38.1	

WHZ- Weight-for-height z-score; BMIZ- Body Mass Index-for-age z-score; WAZ- Weight-for-age z-score; HAZ- Height-for-age z-score.

HIV-positive children also presented with a high burden of undernutrition. A high magnitude (61.9%) of HIV-positive children attending CTCs had moderate to severe stunting. Moderate to severe underweight was also prevalent among 38.7% of children below ten years of age. More than one quarter (26.0%) of HIV-positive children aged between six months and five years presented with moderate to severe wasting. Moderate to severe thinness was prevalent among 21.1% of HIV-positive children aged six years and above. For all types of undernutrition, there were no statistical differences between male and female children.

#### Determinants of undernutrition among HIV-positive children attending CTCs in Tanga

Table 3 shows the results of regression analysis on the factors associated with undernutrition among HIV-positive children attending CTCs with their caregivers. After adjusting for covariates and confounders, a unit increase in age of HIV-positive children (in months) was more likely to be associated with poor linear growth (β = −0.35, p<0.001). Compared to uneducated caregivers, caregivers with at least primary education had a lower chance of having stunted children (β = 0.87, p = 0.035). Household food insecurity was also associated with children's poor linear growth (β = −0.01, p = 0.027). Low feeding frequency was also associated with poor linear growth among HIV-positive children (β = 0.11, p = 0.016). Malaria episodes in the past six months and advanced HIV clinical stage were also associated with poor linear growth among HIV-positive children.

**Table 3:** Regression analyses of factors associated with undernutrition (stunting, wasting, underweight, and thinness) among HIV-positive children.

Variable	Stunting (HAZ-score)				Wasting (WHZ-score)				Underweight (WAZ-score)				Thinness (BMIZ-score)			
	Bivariate		Multivariate		Bivariate		Multivariate		Bivariate		Multivariate		Bivariate		Multivariate	
	Beta	p-value	Beta	p-value	Beta	p-value	Beta	p-value	Beta	p-value	Beta	p-value	Beta	p-value	Beta	p-value
**Age**	−0.32	<0.001	−0.35	<0.001	0.17	0.024	0.20	0.009	−0.04	0.363	−0.06	0.180	−0.27	<0.001	−0.28	<0.001
**Sex**	−0.07	0.071	−0.06	0.076	0.06	0.413	0.06	0.411	0.05	0.308	0.31	0.484	0.06	0.182	0.04	0.353
**Education**																
None	1.00												1.00			
Primary	0.15	<0.001	0.87	0.035	0.16	0.841	0.02	0.808	0.09	0.095	−0.01	0.942	−0.01	0.950	−0.05	0.273
>Primary	0.10	0.011	0.36	0.395	0.11	0.195	0.07	0.451	0.18	0.001	0.07	0.196	0.13	0.008	0.06	0.186
**Wealth index**	0.11	0.002	−0.02	0.606	0.15	0.036	0.09	0.294	0.17	<0.001	−0.01	0.790	0.08	0.053	0.01	0.876
**Food insecurity**	−0.20	<0.001	−0.01	0.027	−0.15	0.038	0.06	0.541	−0.29	<0.001	−0.17	0.003	−0.13	0.002	−0.08	0.134
**Food frequency**	0.18	<0.001	0.11	0.016	0.14	0.054	0.05	0.601	0.25	<0.001	0.12	0.029	0.14	0.001	0.11	0.026
**Dietary diversity**	0.17	<0.001	0.08	0.050	0.08	0.305			0.20	<0.001	0.07	0.165	0.08	0.074	−0.01	0.972
**TB**	−0.07	0.070	−0.06	0.096	−0.04	0.579			−0.09	0.053	−0.07	0.111	−0.07	−0.103	−0.07	0.066
**Diarrhea**	−0.04	0.261			−0.08	0.267			−0.07	0.160	−0.04	0.452	−0.04	0.365		
**Malaria**	−0.71	0.051	−0.09	0.013	−0.17	0.020	−0.12	0.119	−0.16	0.001	−0.14	0.002	0.03	0.438		
**ARI**	−0.05	0.163	0.03	0.350	−0.15	0.051	−0.13	0.120	−0.12	0.008	−0.01	0.826	0.01	0.840		
**HIV stage**	−0.12	0.001	−0.12	0.001	−0.25	0.001	−0.25	0.001	−0.18	<0.001	−0.14	0.001	−0.03	0.461		
**HAART**	0.03	0.479			0.09	0.227			0.03	0.565			−0.11	0.011	−0.12	0.002
**Adherence**	0.05	0.177	0.03	0.423	0.02	0.849			−0.03	0.501			−0.05	0.276		

WHZ- Weight-for-height z-score; BMIZ- Body Mass Index-for-age z-score; WAZ- Weight-for-age z-score; HAZ- Height-for-age z-score; TB- Tuberculosis; ARI- Acute respiratory infection; ART- Antiretroviral therapy.

Wasting was associated with age and HIV clinical stage (Table 3). Younger children were more likely to experience wasting (β = 0.20, p = 0.009), and an increase child's HIV clinical stage from early to advanced was more likely to be associated with severity of wasting (β = −0.25, p = 0.001). Feeding practices were not significantly associated with wasting among children of this population.

Food insecurity, feeding frequency, malaria, and HIV clinical stage were associated with poor weight gain for age after controlling for covariates and confounders (Table 3). HIV-positive children residing in households with food insecurity were more likely to be underweight (β = −0.17, p = 0.003). Low feeding frequency was also associated with poor weight gain for age among HIV-positive children (β = 0.12, p = 0.029). Having malaria in the past six months (β = −0.14, p = 0.002) and advanced HIV clinical stage (β = −0.14, p = 0.001) were associated with poor weight gain among children in this population.

Factors associated with thinness included age in months, feeding frequency, and ART use (Table 3). After adjusting for covariates and confounders, a unit increase in age of HIV-positive children (in months) was more likely to be associated with poor body mass index for age (β = −0.28, p<0.001). Feeding frequency was also more likely to be associated with poor body mass index for age among HIV-positive children (β = 0.11, p = 0.026). HIV-positive children on ART were more likely to have poor body mass index for age (β = −0.12, p = 0.002).

#### Factors associated with feeding frequency

Table 4 shows the results of bivariate and multivariate regression analysis for the factors associated with feeding frequency among the HIV-positive children attending CTCs. Children residing in households with a low wealth index were less likely to have a high feeding frequency (β = 0.06, p<0.001) so were children whose households had high levels of food insecurity (β = −0.05, p<0.001). HIV-positive children whose caregivers had higher than primary school education were more likely to have higher feeding frequency (β = 0.34, p = 0.015), Finally, having acute respiratory tract infections in the previous six months was associated with low feeding frequency among HIV-positive children (β = −0.23, p = 0.001).

**Table 4:** Factors associated with feeding frequency.

Variable	Bivariate regression			Multivariate regression		
	Coefficient	Beta	P-value	Coefficient	Beta	p-value
**Age (months)**	−0.01	−0.05	0.160	−0.01	−0.04	0.201
**Sex**	−0.06	−0.03	0.401	−0.10	−0.05	0.138
**Education**						
None	1.00					
Primary	0.43	0.20	<0.001	0.07	0.03	0.420
>Primary	0.93	0.26	<0.001	0.34	0.09	0.015
**Wealth index**	0.17	0.38	<0.001	0.06	0.14	<0.001
**Food insecurity**	−0.06	−0.49	<0.001	−0.05	−0.39	<0.001
**TB**	0.13	0.03	0.344			
**Diarrhea**	−0.26	−0.13	<0.001	−0.04	−0.02	0.563
**Malaria**	−0.01	−0.01	0.982			
**ARI**	−0.53	−0.26	<0.001	−0.23	−0.11	0.001
**HIV stage**	−0.02	−0.01	0.811			
**HAART**	0.08	0.03	0.459			
**ART adherence**	0.15	0.13	0.001	0.04	0.04	0.286

TB- Tuberculosis; ARI- Acute respiratory infection; ART- Antiretroviral therapy.

### Results of the focus group discussions

We identified seven major themes from the seven focus group discussions that were conducted. The themes evolved from the locally identified determinants of undernutrition and poor feeding practices. They included low feeding frequency and dietary diversity, food insecurity, poverty and low income, and opportunistic infections. These four themes were related to the factors associated with undernutrition in the quantitative survey. Three other themes emerged that were unrelated to the quantitative survey: stigma, lack of nutrition education or misconceptions, and traditions and ways of life.

#### Feeding frequency and dietary diversity

In this study, low feeding frequency was a common practice among participants. Caregivers fed their children twice a day or at the same frequency as adults or otherwise normal children.

“…my children eat only twice a day although I am a food vendor. In the morning I give them porridge until at 4 PM, when they eat the last meal of a day. We skip lunch because we cannot afford it. I prefer giving the last meal closer to night just to make them survive.” (A 26-year-old food vendor and mother of two HIV-positive children)

Participants explained that when they increased their children's feeding frequency, their nutrition statuses improved.

“My child's hair was so weak, so thin, and she had a large abdomen and thin legs. I was told that she had kwashiorkor. This was when her biological mother had just died. She was checked for worms, and treated for it, also given Septrin (an antibiotic given routinely for HIV-positive children). I started to give her more food, many times a day. As a result, she started to thrive. She was later started on ARV, and now she looks healthy, all symptoms of kwashiorkor are gone.” (A 28-year-old caregiver and mother of four children)

Increasing feeding frequency also showed improvements in micronutrients profiles. It happens when the quality of diets is improved among children living with HIV/AIDS.

“I did not feed my child five times a day. Instead, I gave her three times just like the rest of the family including adults. When I came to hospital, she was found to have low weight, failed to grow, and had low blood contents. I then started to feed actively, especially fruits, vegetables, mixing porridge with peanuts…. she started to grow healthy, her blood contents increased, and weight too, even other diseases had stopped.” (A 28-year-old married woman, mother of three children, one of whom is HIV-positive)

HIV-positive children in Tanga are fed with fewer food types. Caregivers pointed to a lack of money to buy other types of food although such foods are available in the markets.

“…nowadays, we cook only once a day, either plain corn porridge or stiff porridge, so the child eats only what is available in the morning until evening. If we are lucky to have something to eat at night, we give her otherwise we sleep hungry. We are taught to give them mixture of eggs, meat, milk, and vegetables. Although these foods are available, we can not afford them.” (A 50-year-old caregiver, mother of four children and a small-scale farmer)

Caregivers may be given nutrition education on how to mix foods for their children. But many of them still consume a mixture of similar types of foods unknowingly. These foods have similar nutrition contents and might not provide added benefits, no matter the number of items included or frequency at which they are fed.

“…in her porridge, I mix flour from corn, millet, sorghum, rice, and other cereals. I was advised to mix a variety of foods to improve nutrition. This porridge I give many times in a day.” (A 24-year-old mother of two who is also an entrepreneur, selling a similar mixture to her peers)

When asked who taught her this mixture, she said, “This is what I was taught by my nurses here.”

Giving high dietary diversity can improve nutrition status and even physical body functions. Several caregivers explained that their children had improved after they received nutrition advice to increase diversity.

“My child had poor health, weak, and very thin when I started her in this clinic. She could not stand up by herself or play, although she had the age of a child who could walk and run. She started ARV, and I was given nutrition advice, which I followed, I started to give milk, adequate volumes of various foods, and fruits everyday. Her health status improved, and gained weight. She could then play and walk.” She went on and say, “I have stopped giving that quality foods now because, now I can not afford it… we cook only once for what we have…I cannot give milk, I can't even cook with cooking oil…as a result, she is falling back so fast.” (A 30-year-old farmer and caregiver of seven children)

#### Food insecurity and hunger

The HIV-positive participants mentioned that low food availability in their households is common because they cannot do manual farm work due to their health condition. They have low purchasing power for foods despite being available in the markets. As a result, they cannot have the recommended feeding frequency and dietary diversity. Ultimately, their children suffer from undernutrition.

“I am a farmer, and depend on agriculture. Last year for example, it did not rain enough. We did not get harvests, especially corn. Foods were little and less diverse…we did not have income, and could not even buy enough foods from markets. This child ate less and lost weight. When this child is sick with fever, he wants to eat some good foods; it was impossible for him to get it. He did not want to eat stiff porridge available all day, everyday.” (A 56-year-old grandmother of an HIV-positive child)

“For farmers like me, sometimes, we do not harvest enough because we depend on rain and can do it manually with hand hoe, so we cannot cultivate enough because of our health. Our yields are low and we are in a constant food shortage.” (A 59-year-old widow and farmer)

#### Poverty and low income

Participants pointed to poverty and low income as an underlying cause of poor feeding practices and undernutrition among HIV-positive children in this region.

“..now life has become so difficult. I am sick, and cannot do hard work, especially farming. It causes children to lose weight because good food requires a lot of money. I do not have income. I know that milk is important especially when you use strong medications like ARVs, but, I can stay one year before I or my child could drink it. Sometimes in my family we sleep without eating anything… my child goes to school without breakfast. When I am well I go to work, and get paid 2000 shillings for example, I can only afford to buy basic cheap food. That is why children are undernourished.” (A 50-year-old mother of four children)

“…low income is the reason for undernutrition. I struggle to look for money, the child eat only twice, in the morning and then at evening even when I myself sell food and fruits…when my husband died, he left us a little money. My family income suddenly dropped. I am the only person who has to provide for my family and it is not enough.” (A 37-year-old mother of two)

#### Opportunistic infections

HIV/AIDS suppresses immunity leading to opportunistic infections. Participants in this study gave examples where undernutrition of their children resulted from such infections.

“My child had swellings on his neck. During the time of this illness, he was not eating well, and complained of painful swallowing. He became wasted, like those people who are real sick of AIDS. But after medication (not ARVs), he improved; now he eats well and his weight gained.” (A 31-year-old mother of two children)

Tanga is also an endemic area for malaria transmission. Participants also explained how malaria contributed to their children's poor nutrition status.

“This week, my child was sick of malaria, she could not hold food, she has fever, and she also has oral curd-like condition they say is fungus. She has pain when eating and has lost too much weight… her suffering gives me depression.” (A 32-year-old mother of two HIV-positive children)

#### Stigma related to HIV status and child undernutrition

Participants indicated that stigma against people living with HIV/AIDS can lead to a child's poor health including undernutrition. For example:

“Another problem is stigma. Colleague of my child tells her at school that she has AIDS… this created stigma. As a result, she lost hope at first then refused to eat; at the end she lost weight. She told me that her friends did not want to play with her. People in our society stigmatize our children and us. Even if we feed them with whatever food available, the stigma they face weakens them physically and psychologically.” (A 24-year-old entrepreneur and mother of two HIV-positive children)

“…another reason is stigma especially for those who are older children attending schools. They are isolated and mocked. They feel weak and lose hope. Their feeding deteriorates even in the presence of enough food as well.” (A 20-year-old mother of one HIV-positive child)

Stigma also exists within families. A 30-year-old housewife and mother to two while caring for her late sister's HIV-positive child said:

“My husband leaves money for his children's food only, not for this HIV-positive one. Even when I leave some for this affected child, he does not like it, and get mad at me.”

#### Lack of nutrition education or misconceptions, and traditional beliefs

In the current study, caregivers had low levels of nutrition knowledge especially of adequate feeding frequency, proper dietary diversity, and mixing of foods for young HIV-positive children.

“Majority of us do not understand what is a good quality food. Education on nutrition is lacking…we have our myths from our traditions. We thought that artificial juice had equal nutrition values as natural juice based on fruits available in our environment, such as pawpaw, mangoes, oranges, banana, etc. I would like to know how to make them. With such knowledge, I believe, I will be able to make right decisions on foods. All quality food substances are available I believe, I just need to understand how to combine them in to a quality diet. For example, if I cannot afford meat, what else should I replace with? What cheap options can I substitute with?” (A 33-year-old father of four children)

A 19-year-old participant thinks that vegetables are not ‘good food’ as it is the food of poor, and said “I eat vegetables a few days only when I do not have money for alternatives like red meat.” She added, “Good food is anything other than green vegetables. Vegetables are not considered as good food here. In our normal diet we do not eat them, even when available.”

## Discussion

HIV-positive children attending CTCs had poor nutrition status despite vast food availability in the Tanga region. For example, 61.9% of all recruited HIV-positive children were stunted; 38.7% of HIV-positive children below ten years were underweight; 26.0% of HIV-positive children between six months and five years had wasting; and 21.1% of HIV-positive children above five years were thin. Feeding practices of HIV-positive children attending HIV care and treatment centers were poor. Among the children recruited to participate in this study, 88.1% had a feeding frequency below the recommended value of five times a day. About two thirds of the children had dietary diversity scores of three or below. Triangulated evidence of the quantitative and qualitative studies showed that the poor feeding practices in Tanga region were associated with undernutrition in this region. Regression analyses of factors associated with different types of undernutrition pointed to feeding frequency as a common risk factor for stunting, underweight, and thinness. This is the first study to demonstrate the high magnitudes of poor feeding practices among HIV-positive children in areas known to have adequate food production and its association with undernutrition.

Magnitudes of poor nutrition status in this food rich region among HIV-positive children was higher compared to the general population [19]. For example, while 49.0% of children in the general population were stunted, 61.9% of the HIV-positive children in this study were stunted. Similarly, for all other types of undernutrition, magnitudes were higher in this study than in the general population. Contrary to our prior thoughts, magnitudes of undernutrition among HIV-positive children in this food secure region were also higher compared to those of ART-treated HIV-positive children in another city in Tanzania [28]. As in our previous study [28], ART did not have a statistically significant influence over nutrition status of most types of undernutrition.

This study found an inverse association between age in months and linear growth. Stunting is a chronic form of undernutrition resulting from the cumulative effects of food shortages and poor feeding practices [56]. Compared to other forms of undernutrition, stunting is an irreversible condition. Its effects are long term and can result in poor brain and intellectual development. Our results are different from another study we conducted in another city in Tanzania [28]. Results of the qualitative study showed that low feeding frequency, low amounts, and limited variety of feeding were also common and contributed to poor nutrition statuses.

Low feeding frequency was associated with stunting, underweight, and thinness among HIV-positive children in this study. Similar conclusions were made for stunting, wasting, and underweight in our previous study among HIV-positive children in a different city in Tanzania [28]. WHO recommends a feeding frequency of at least five times a day to provide for the high energy and nutrients demand among HIV-positive children [22]. Improvement in feeding frequency and other feeding practices can bring about positive changes in nutrition status among children in the general population [4]. Our study strengthens the need for improvement in feeding practices, particularly feeding frequency, in the fight against undernutrition.

In this study, overall feeding practices of HIV-positive children were poor, despite food production being high in the region. Households of HIV-positive children also had high levels of food insecurity; about three quarters of the children resided in households with moderate to severe food insecurity. Caregivers of HIV-positive children, in particular, may therefore not be able to provide enough food for their families even if general food availability in the area is good [21]. As in other regions in Tanzania [28], such caregivers may succumb to socio-economic disadvantages and are also prone to a high burden of opportunistic infections as they are also more likely to be HIV-positive. The main sources of food in this region are small-scale farming and animal husbandry. Therefore, poor families with no or small farmlands, or HIV-positive people who are not strong enough to do the manual labor required on a farm, may have less food for their households. This in turn may lead to a chronic shortage of food and may bring about low dietary diversity and inadequate intake of nutrients [10], [11].

Feeding frequency might also be affected in a similar way. In this study, an independent association was detected between feeding frequency among households affected by food insecurity and poverty. Results from focus group discussions among caregivers of HIV-positive children also showed that households with food insecurity were more prone to low feeding frequency of their children. Because of the lack of food, children ate twice a day on most occasions. Similar results were observed in Ethiopia among adolescents with unknown serostatus [57] and HIV-positive adults.[58] However, these areas have been known to have long periods of hunger and food insecurity, characteristics that differ from our study.

Feeding practices, and in particular feeding frequency, were also associated with the caregiver's education level. In this case, even when food is available at the household level and other factors are controlled for; caregivers with low education levels may not provide foods to their children at the recommended frequency. More than one fifth of the caregivers of HIV-positive children in this population were not educated. In the qualitative study, too, caregivers had little knowledge of nutrition and how to feed their children. There were many misconceptions based on local beliefs and traditions that also led to wrong choices of foods. The level of education may determine nutrition knowledge [59]. When caregivers are given such knowledge, they improve feeding practices, including feeding frequency and dietary diversity, of their children [3], [58], [60], [61].

Poor feeding practices were associated with a burden of other diseases. For example, low feeding frequency was associated with the chance of having had acute respiratory tract infections at the same time. Such acute diseases are especially common among children with HIV/AIDS and undernutrition [25]. Results from our qualitative study may help to explain this phenomenon. Because of low immunity, HIV-positive children who are also affected by undernutrition are prone to other infections including upper respiratory tract infections. Children with these conditions improved upon proper feeding in adequate amount and frequency.

The results of this study should be interpreted with care owing to the following limitations. First, the nature of our cross-sectional design limits our conclusions on causality. For example, the direction of associations between feeding practices and infections such as respiratory tract infections cannot be ascertained. However, triangulation of the quantitative and the qualitative study can help further explore such associations, though it is not sufficient to ascertain causality. Previous models have also suggested the association between diseases such as malaria and respiratory tract infections with undernutrition is possibly through poor feeding practices among HIV-positive children [62]. Second, this study may be subjected to recall bias based on self-reporting by caregivers of HIV-positive children on items such as feeding practices, food insecurity, and other demographic characteristics. Third, our findings may not be generalizable beyond the settings of high food productivity. Evidence already exists on the high magnitude of poor feeding practices among HIV-positive children in general settings [15] and in food insecure areas among HIV-negative [57] and HIV-positive adults [58]. Fourth, the reliability of our measurement for ART adherence may be questionable, as it was not validated in scientific articles from the settings of this study. However similar method is being used in CTC clinics to monitor ART adherence and conduct adherence counseling. Fifth, we did not translate the translated script from Swahili to English, to the original Swahili language to compare with the original script. This could have lead to the unintended translation. However, the first author who supervised the overall translation process is fluent both in English and Swahili language and tried to avoid the fatal misunderstandings.

## Conclusion

In conclusion, HIV-positive children attending care and treatment centers in Tanga, a region with adequate food production, had poor nutrition statuses and low feeding frequency and dietary diversity scores. Low feeding frequency was associated with stunting, underweight, and thinness. Factors associated with low feeding frequency included low wealth index, low maternal or caregiver education, household food insecurity, and acute respiratory tract infections in the past six months.

To ameliorate the high burden of undernutrition among HIV-positive children, feeding practices, in particular feeding frequency, should be improved even in areas where food production is high and food is available at low cost. To achieve this, more focused interventions may be necessary to help HIV-positive children in those households that face high food insecurity, poverty, and low education of caregivers. Nutrition training of health workers can help provide this support, reduce misconceptions regarding healthy foods [63], and help caregivers utilize efficiently and well the vast food availability in the region [3]. Nutrition counseling may also teach methods of food storage households who are affected by food insecurity but live in food rich regions [22]. Frequent nutrition counseling can help to guide such families into proper use of available resources including foods and give priorities to children with high nutritional demand.
